# Evaluation of facial soft tissue thickness in asymmetric mandibular deformities after orthognathic surgery

**DOI:** 10.1186/s40902-021-00323-5

**Published:** 2021-10-09

**Authors:** Luo Huang, Zhicong Li, Jing Yan, Lunqiu Chen, Zheng-guo Piao

**Affiliations:** Department of Oral and Maxillofacial Surgery, Affiliated Stomatology Hospital of Guangzhou Medical University, Guangzhou Key Laboratory of Basic and Applied Research of Oral Regenerative Medicine, 39 Huangsha Avenue, Guangzhou, 510150 NO China

**Keywords:** Orthognathic surgery, Skeletal Class III malocclusion, Facial asymmetry, Soft tissue

## Abstract

**Objectives:**

The purpose of this study was to compare differences in facial soft tissue thickness in three-dimensional (3D) images before and after orthognathic surgery in patients with skeletal Class III malocclusion and to obtain a better understanding of the relationship between hard and soft tissue changes after surgery.

**Materials and method:**

The present retrospective study included 31 patients with skeletal Class III malocclusion with mandibular chin deviation greater than 4 mm who had undergone cone-beam computed tomography before and 6 months after surgery. Seven bilateral points were established. Measurements were taken from software-generated multiplanar reconstructions. The predictor variables were timing (pre- and postoperatively) and side (deviated vs. nondedicated). A regression model and correlation analysis were conducted for statistical analysis.

**Results:**

The difference of bilateral facial soft tissue thickness was statistically significantly different between deviated and nondeviated sides (*P* < 0.05), with lower values observed on the deviated side. The soft tissue thickness has become nearly symmetric at local regions of the lower thirds of the face after orthognathic surgery. However, most measurements showed a negative correlation between changes in soft tissue thickness and changes in bone tissues.

**Conclusions:**

Skeletal Class III malocclusion with facial asymmetry is accompanied by differences in soft tissue thickness when comparing Dev and N-Dev sides of the posterior region of the mandible, where soft tissues are thinner on the Dev side. Soft tissue thickness can compensate for or camouflage the underlying asymmetric mandible. In addition, the asymmetric soft tissue thickness on the lower third of the face can be partially improved by orthognathic surgery, but the amount of soft tissue thickness change is not consistent with that of hard tissue positional change.

## Background

Facial asymmetry is common among patients who desire orthognathic surgery. According to reports, facial asymmetry mainly occurs in the mandible, which is diagnosed in almost 50% of patients with skeletal Class III malocclusion [1–3]. Although orthognathic surgery has been successful in changing the position of the mandible and increasing facial contour symmetry, there are still some skeletal asymmetries after the surgery [4, 5]. This residual asymmetry is due to the lack of preoperative analysis of soft and hard tissue asymmetry, and the difficulty of using individual features of soft tissue to predict soft tissue changes. In addition, the reasons for the differences in individual soft tissue responses are very complex and not yet fully understood. In theory, soft tissues cover bony structures, and changes in hard tissues can directly affect soft tissues. However, soft tissues respond differently to the anterioposterior movement of the maxilla or mandible [6, 7]. The degree of the asymmetry of soft tissue is more than that of hard tissue. This fact shows that even if the hard tissue is perfectly corrected, the symmetry of soft tissue cannot be guaranteed after orthognathic surgery. Therefore, the positioning of the facial asymmetry tissues and the determination of the relationship between the asymmetry of the soft and hard tissues have become important factors for detailed diagnosis and precise treatment plan.

Facial asymmetry is probably associated with bony tissues, soft tissues, or both [7]. A symmetric and balanced face may have skeletal asymmetry, but this asymmetry will be compensated for or masked by soft tissue [8]. Asymmetric soft tissue can be found in individuals with symmetric hard tissue [9], while patients with clinical symmetry or mild asymmetry may show severe skeletal asymmetry in X-ray analysis [10]. However, there are still few studies detailing the role of each tissue in facial asymmetry. Additionally, whether preoperative and postoperative soft tissue asymmetry accurately reflects the contour of hard tissue is unclear. It can be assumed that the thickness of facial soft tissues affects the visual perception of symmetry by disguising or compensating for potential skeletal asymmetry, showing different thicknesses on the bilateral sides of the face. However, a three-dimensional (3D) quantitative analysis of soft tissue thickness changes in orthognathic surgery has not been thoroughly studied. Only Lee et al. [11] performed a 3D study of chin soft tissue changes after orthognathic surgery in patients with deviated mandibular prognathism. Therefore, the purpose of this study was to compare the differences in facial soft tissue thickness in 3D images before and after orthognathic surgery in patients with skeletal Class III malocclusion and to obtain a better understanding of the relationship between hard and soft tissue changes after surgery.

## Materials and methods

### Study and subjects

This study included 31 adult patients with skeletal Class III malocclusion and facial asymmetry who underwent conventional bimaxillary orthognathic surgery (1-piece Le Fort I and bilateral sagittal split ramus osteotomy (BSSRO)) with presurgical orthodontic treatment from October 2018 to November 2019 at the Department of Oral and Maxillofacial Surgery of Guangzhou Medical University Stomatological Hospital. The demographic and radiographic data of patients are presented in Table 1. The mandibular setback mount was measured at the pogonion. The setback amount range was 2–8 mm, and the average was 4.89 mm (right, 5.14 mm; left, 4.27 mm). The gonial angle decreased by 6.07 ± 4.47° (right, 7.33°; left, 5.73°). This study was approved by the Research Ethics Committee of Guangzhou Medical University Stomatological Hospital. Written informed consent was obtained from all patients before the initiation of treatment.

**Table 1:** Demographic and radiographic data of patients in the sample

Characteristic	Value
Patients, *n*	31
Male	10
Female	21
Age at operation, *y*	26.6 ± 6.2
Me deviation (mm), mean±SD (before surgery)	7.7 ± 3.4
Me deviation (mm), mean±SD (after surgery)	2.0 ± 1.2

The inclusion criteria were as follows: (1) patients older than 18 years of age; (2) patients with skeletal Class III malocclusion with obvious mandibular asymmetry, showing that the chin (menton) deviated more than 4 mm from the midline of the face; and (3) patients for whom serial cone-beam computed tomography (CBCT) images obtained before and after surgery were available. The exclusion criteria were as follows: (1) patients with existing congenital craniofacial deformities, such as cleft lip or palate; (2) patients who had undergone mandibular contour trimming or any other supplementary surgery; (3) patients with a history of maxillofacial trauma; and (4) patients for whom images were of insufficient quality due to motion blur artifacts.

### CBCT and reconstruction

CBCT images were obtained with a NewTom scanner (Imaging Science International, Hatfield, PA, Italy) using a 200–400-mm field of view, 120 kVp, and 47.7 mA, resulting in a 0.4-mm voxel size. All patients received CBCT before (T1) and 6 months after (T2) surgery, during which the lips were in a relaxed position. The CBCT images were converted to DICOM 3.0 files and evaluated with the use of ProPlan CMF 3.0 (Materialise, Leuven, Belgium). The thresholds were defined as 226 to 3071 Hu for hard tissues and 700 to 225 Hu for soft tissues.

### 3D measurement

**Fig. 1:**
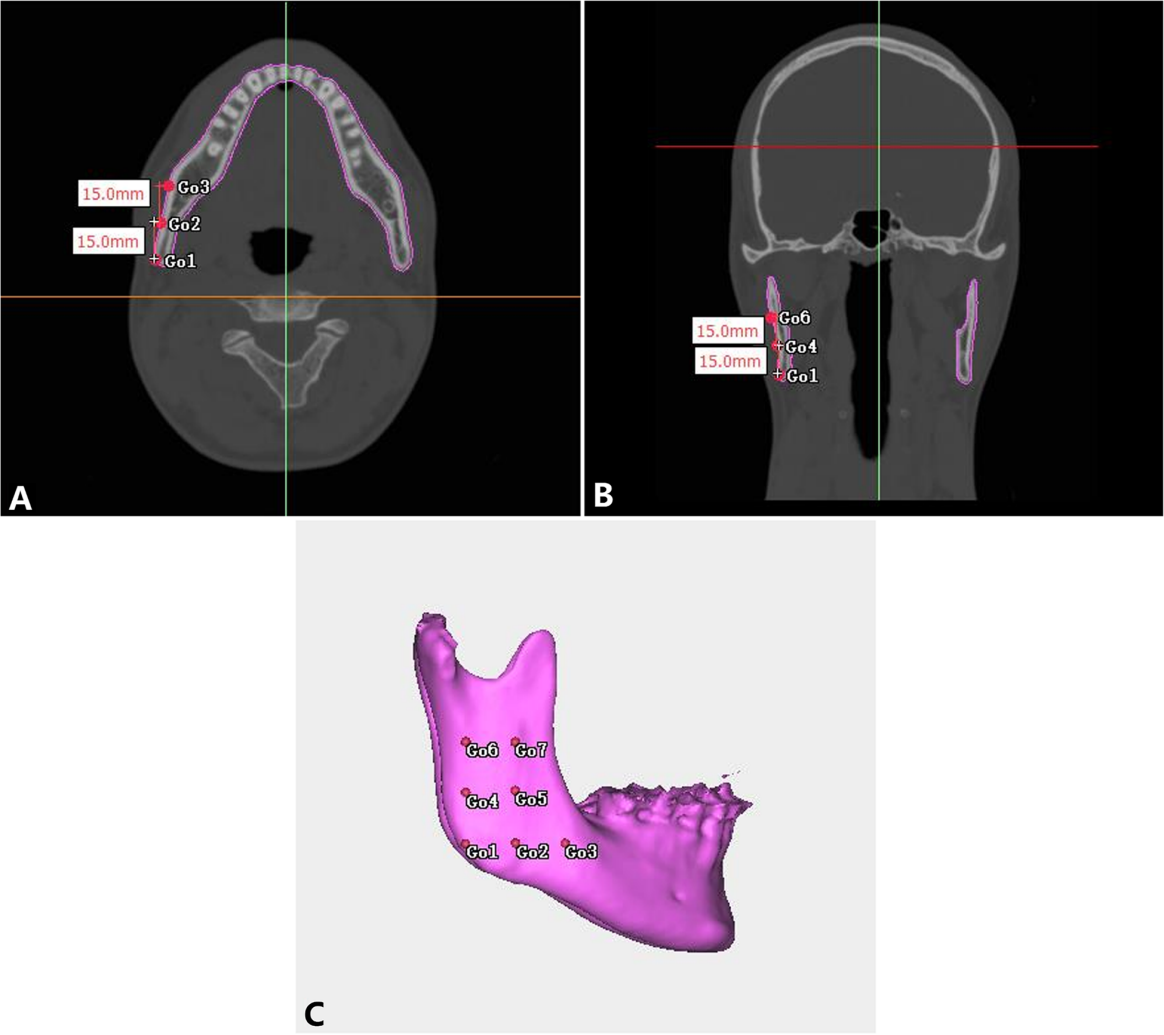
The 3D position of the points used in this study. **A** Axial slice with points Go1, Go2, and Go3; **B** coronal slice with points Go1, Go4, and Go6; **C** lateral view of 3D reconstruction illustrating the position of the reference points used to measure hard tissue and soft tissue asymmetry and soft tissue thickness on the right side

**Fig. 2:**
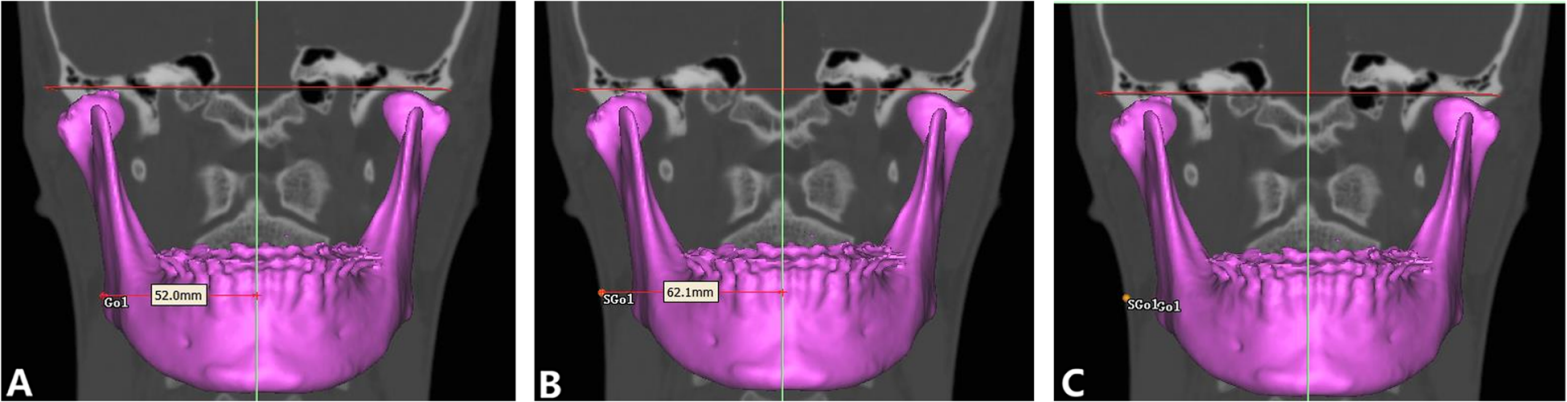
Hard and soft tissue distance and soft tissue thickness were measured by establishing the horizontal line from the midsagittal reference plane to reference points on hard and soft tissue surfaces. **A** Hard tissue distance (Hard-D); **B** soft tissue distance (Soft-D); **C** soft tissue thickness (Soft-Th)

Two reference planes were used to redirect the image: the Frankfort horizontal plane (FH), which was constructed from bilateral orbitals and left porion, and the midsagittal plane (MSP), which passed through the nasion and sella and perpendicular to FH. The side of the lateral menton deviation associated with MSP was defined as the deviated side (Dev side), and the opposite side was defined as the nondeviated side (N-Dev side). Seven 3D landmarks were selected according to the procedure used in a previous study [12]. The initial measurement point used for the evaluation of mandibular soft tissue was gonion (Go) and was marked on the 3D image and then accurately adjusted on the multiplanar reconstructions and recognized as point Go1. Then, extending from Go1 to the corresponding soft tissue surface was defined as point SGo1. To cover the masseter muscle as large as possible, six additional points deriving from Go1 and SGo1 were produced (Table 2; Fig. 1). The measurement method of hard and soft tissue distances, as well as soft tissue thickness, was based on the method used in a previous study [12] (Table 2). The amount of asymmetry was calculated by using the automatic measurement function of the software (Fig. 2). The 3D cephalometric analysis was performed on all samples by a single investigator (L.H.).

**Table 2:** Definition of landmarks and measurements used in this study

	Definition
Point	
Go1	Gonion point, the midpoint on the mandibular angle between the ramus and the body
Go2	Point located 15 mm in front of the Go1 on the same axial slice as Go1
Go3	Point located 15 mm in front of the Go2 on the same axial slice as Go1 and Go2
Go4	Point located on 15 mm above the Go1 shown on the coronal slice
Go5	Point located 15 mm in front of the Go4 on the same axial slice as Go4
Go6	Point located on 15 mm above the Go4 shown on the coronal slice
Go7	Point located 15 mm in front of the Go4 on the same axial slice as Go6
Measurement	
Hard tissue distance (Hard-D)	Horizontal distance between the midsagittal plane and the outer cortex of the bone
Soft tissue distance (Soft-D)	Horizontal distance between the midsagittal plane and the soft tissue surface
Soft tissue thickness (Soft-Th)	Horizontal distance between the outer cortex of the bone and the soft tissue surface

### Statistical analysis

Statistical analysis was performed with SPSS 13.0 for Windows. The means, standard deviations (SDs), and frequencies were calculated. To examine intraobserver and interobserver errors, we randomly selected 15 patients, and all linear measurements were performed bilaterally on 2 occasions, at the initial assessment and at the reassessment after 10 days, by a single observer (a graduate student). The intraobserver reliability and interobserver reliability for the first and second assessments were analyzed on the basis of the intraclass correlation coefficient (ICC). A paired *t* test was used to compare the Dev side and N-Dev side soft tissue thickness, as well as preoperative and postoperative changes. Spearman correlation analysis was used to evaluate the relationship between soft and hard tissue changes and soft tissue thickness.

## Results

ICCs ranged from 0.907 to 0.969 for intraobserver reliability and from 0.905 to 0.921 for interobserver reliability, indicating a high reliability of the measurements used in this study.

### Facial soft tissue thickness and the differences between two sides before and 6 months after surgery

The thickness of Dev side soft tissues before orthognathic surgery was thinner than that of N-Dev side soft tissues. The difference in bilateral facial soft tissue thickness was statistically significant between the Dev and N-Dev sides (*P* < 0.05, Table 3). The thickness of the soft tissue on the Dev side increased obviously at 6 months after surgery (Table 3). The thicknesses of bilateral soft tissues at the Go3, Go4, and Go6 points were significantly different at T1, but no significant difference was observed at T2. Moreover, although there was a significant change from T1 to T2, the bilateral differences at Go1, Go2, Go5, and Go7 remained statistically significant (*P* < 0.05, Table 3).

**Table 3:** Comparison of facial soft tissue thickness between deviated (Dev) and nondeviated (N-Dev) sides before (T1) and 6 months (T2) after surgery

Variable	T1				T2				T1–T2
	Dev	N-Dev	Difference	***P*** value^†^	Dev	N-Dev	Difference	***P*** value^†^	***P*** value^‡^
Go1	9.67 ± 3.11	11.00 ± 3.74	1.34 ± 1.31	0.000	12.50 ± 4.63	13.66 ± 4.99	1.16 ± 0.96	0.000	0.000
Go2	13.87 ± 2.89	14.69 ± 2.93	1.17 ± 0.88	0.000	15.98 ± 3.96	16.79 ± 3.99	0.80 ± 1.27	0.002	0.000
Go3	16.07 ± 0.93	17.47 ± 4.54	1.40 ± 1.58	0.000	21.45 ± 4.42	21.97 ± 4.21	0.52 ± 1.33	0.058	0.000
Go4	14.61 ± 2.75	15.82 ± 3.11	1.22 ± 1.35	0.000	15.60 ± 4.25	15.95 ± 4.06	0.34 ± 1.05	0.098	0.146
Go5	18.21 ± 2.65	19.09 ± 2.50	0.87 ± 0.92	0.000	20.20 ± 3.66	20.87 ± 3.69	0.67 ± 1.04	0.002	0.000
Go6	16.35 ± 2.54	17.70 ± 2.53	1.35 ± 1.35	0.000	16.18 ± 3.14	16.52 ± 3.15	0.34 ± 1.19	0.14	0.032
Go7	19.93 ± 2.19	20.99 ± 2.04	0.28 ± 0.55	0.000	21.12 ± 3.15	21.40 ± 3.098	0.28 ± 0.55	0.013	0.004

^†^*P* values were calculated by paired *t*-test. ^‡^Difference between T1 and T2 was calculated by paired *t*-test

### Correlation between hard and soft tissue changes after surgery

Correlation analysis was used to study the relationship between hard tissue and soft tissue changes after surgery. The thickness of most mandibular soft tissues was positively correlated with the change in soft tissue distance (*P* < 0.05). However, most measurements showed a negative correlation between changes in soft tissue thickness and changes in bone tissues (Table 4).

**Table 4:** Correlation between hard and soft tissue changes

	Correlation coefficient (*r*)													
	Contralateral side							Deviated side						
Variable	Go1	Go2	Go3	Go4	Go5	Go6	Go7	Go1	Go2	Go3	Go4	Go5	Go6	Go7
Hard-D/Soft-D	0.218	0.161	0.331	0.309	0.209	0.311	0.194	0.282	0.340	0.315	0.082	0.170	0.290	0.105
Soft-Th/Hard-D	−0.280^*^	−0.526^**^	−0.604^**^	−0.318	−0.401^*^	−0.395	−0.451^*^	−0.549^**^	−0.542^**^	−0.505^**^	−0.360	−0491^*^	−0.547^**^	−0.457^**^
Soft-Th/Soft-D	0.806^**^	0.660^**^	0.171	0.660^**^	0.531^**^	0.373	0.530^**^	0.510^*^	0.498^*^	0.390	0.595^**^	0.348	0.436^*^	0.495^*^

Data are presented as Spearman’s correlation coefficient (*r*) between measurements

^*^Statistically significant, *P* < 0 .05

^**^Statistically significant, *P* < 0 .01

## Discussion

With the increasing amount of attention given to facial appearance, the characteristics of soft tissue have become very important, which has prompted researchers to study the relationship between the symmetry of soft tissue and hard tissue. The thickness of soft tissues can affect the symmetry or asymmetry of facial appearance. A study found that 80% of skeletal asymmetries from the front view can be reduced to 56% in soft tissue thickness in Class III malocclusion, which indicates that soft tissues have the effect of masking the asymmetry of the hard tissues [9]. Young’s [10] study on the relationship between soft tissue morphology and bone structure represented that there was a high covariance between the soft and internal hard tissues. In our study, facial soft tissue thickness was evaluated in individuals with skeletal Class III malocclusion and facial asymmetry. The reference mark proposed in this method is located in the area corresponding to the masseter muscle because this muscle may demonstrate a difference between Dev and N-Dev sides that affects tissue thickness and facial asymmetry [13, 14]. According to our results, statistically significant differences were demonstrated for all points, and the thickness of Dev side soft tissues before orthognathic surgery was thinner than that of N-Dev side soft tissues, which implied that an asymmetric mandible is accompanied by different soft tissue thicknesses. This result was in accordance with Lee’s study evaluating individuals with facial asymmetry and mandibular prognathism [11]. However, Lima et al. [12] determined that asymmetric faces had similar soft tissue thickness on both sides. A possible explanation for the different results would be sample difference. Individuals with skeletal Class III malocclusion and facial asymmetry were included in our research, while all individuals with facial asymmetry were included in Lima’s [12] study. This difference may have affected the results, suggesting that the muscles of the anteroposterior mandible act differently in an attempt to disguise facial asymmetry.

Facial asymmetries are usually corrected by traditional orthognathic surgery. Although orthognathic surgery greatly improves the facial contours of patients with facial asymmetry, some asymmetry will still remain after surgery, which is attributed to the lag in soft tissue reconstruction. Clinically, it is difficult to predict changes in soft tissue before and after orthognathic surgery because there are a wide variety of factors that affect these changes, such as soft tissue thickness, muscle elongation, mastication preference, and facial patterns [15, 16]. In this regard, there are some debates about the factors that contribute to postoperative soft tissue outcome. Some studies have shown that changes in soft tissue have nothing to do with the movement of bone tissue even though surgery has improved the severe asymmetry of the hard tissue. Compared with the movement of the mandibular body, the soft tissue marks on the lip margin of the lower lip were more affected by muscle condition [10]. Additionally, the similarity index of soft tissue in the lower face did not significantly change after orthognathic surgery [17]. However, the results of Joss’s 3D research showed that mandibular retraction surgery can cause the lower lip thickness to increase in patients with mandibular asymmetry [18]. The anteroposterior movement of the mandible would cause changes of soft tissue contour at the symphysis region [19]. To better understand the correlations in the amount of asymmetry between hard and soft tissue on the lower third of the face, surgical changes in the hard and soft tissues were also investigated. Although the thickness of soft tissues increased at 6 months after orthognathic surgery, the soft tissue thicknesses were symmetric only at points Go3, Go4, and Go6. It can be assumed that these points may be located in the area of the masseter muscle with the least strength and thus could be affected more easily by surgery, which would explain why this difference was changed significantly at these sites by surgery. The present study showed a negative correlation between changes in soft tissue thickness and changes in bone tissues, which might be due to the attachment sites of the masseter muscle being stripped during BSSRO and soft tissue thickness changes probably related to muscular tension rather than a direct muscular change. This study has limitations in that other factors influencing soft tissue thickness, such as skeletal morphology, were excluded from the evaluation. Therefore, further research is needed to explain the impact of orthognathic surgery on changes in soft tissue thickness.

## Conclusion

On the basis of these results, we conclude that skeletal Class III malocclusion with facial asymmetry is accompanied by differences in soft tissue thickness when comparing Dev and N-Dev sides of the posterior region of the mandible, where soft tissues are thinner on the Dev side. Soft tissue thickness can compensate for or camouflage the underlying asymmetric mandible. In addition, the asymmetric soft tissue thickness on the lower third of the face can be partially improved by orthognathic surgery, but the amount of soft tissue thickness change is not consistent with that of hard tissue positional change. It is expected that these results can be effectively applied to predict the postoperative outcome of orthognathic surgery.

## Data Availability

The data sets supporting the results of this article are included within the article and its additional files.

## Abbreviations

CBCT: Cone-beam computed tomography
BSSRO: Bilateral sagittal split ramus osteotomy
HU: Hounsfield units
FH: Frankfort horizontal plane
MSP: Midsagittal plane
ICC: Intraclass correlation
Go: Gonion
